# Third Repeat Microvascular Reconstruction in Head and Neck Cancer Patients Aged 65 Years and Older: A Longitudinal and Sequential Analysis

**DOI:** 10.1038/s41598-017-15948-8

**Published:** 2017-11-16

**Authors:** Jonas Löfstrand, Kai-Ping Chang, Jennifer An-Jou Lin, Charles Yuen Yung Loh, Hsuan-Yu Chou, Huang-Kai Kao

**Affiliations:** 1 Department of Plastic Surgery, Sahlgrenska University Hospital, Gothenburg, Gothenburg, Sweden; 2 Department of Otolaryngology-Head & Neck Surgery, Chang Gung Memorial Hospital & Chang Gung University College of Medicine, Taoyuan, Taiwan; 3 Department of Plastic and Reconstructive Surgery, Chang Gung Memorial Hospital & Chang Gung University College of Medicine, Taoyuan, Taiwan

## Abstract

Performing a sequential third free flap for reconstruction of a head and neck defect after cancer resection can be challenging, and the problem is further compounded in elderly patients. The outcomes in this clinical scenario are currently unknown and this study aims to compare the results in elderly patients with younger patients in a high-volume microsurgical unit. A retrospective review of 126 consecutive patients who had undergone three sequential free flap reconstructions after head and neck cancer was performed. The patients were divided into two groups – older or younger than 65 years old (n = 105 and n = 21, respectively). Patient demographics, intraoperative and postoperative outcomes were noted and analyzed. The overall flap success in this patient cohort was 94.4% (7 flap losses in 126 patients). Cardiovascular complications were significantly more common in the older group (19% vs. 1.9%, p = 0.001). Delirium occurred more frequently in the older group compared with the younger group (23.8% vs. 6.7%, p = 0.023). There were no significant differences regarding surgical complications. With adequate planning, a sequential third free flap can be performed safely and successfully in patients who are more than 65 years of age. Particular attention to the perioperative morbidity in elderly patients is crucial for successful outcomes.

## Introduction

Head and neck (H&N) cancer is a major global health issue and the seventh most common type of cancer worldwide, with an annual estimated incidence of 600,000 cases^1^. In Taiwan, H&N cancer has been one of the top 10 leading causes of cancer death consistently. Advancement in modern medicine results in prolonged life expectancy, thus increasing percentage of elderly population. In the year 2010, 10.7% of Taiwan’s population was over 65 years of age. The majority of H&N cancers occur during the fifth and sixth decades of life, with more than 20% of patients over 70 years of age^2^. Even though several subsites of H&N cancer is primarily treated by radiation, surgery remains to be the most primary treatment modality for most H&N cancers irrespective of age, and the course of surgical intervention may be combined with adjunctive radiotherapy and/or chemotherapy^3^. With a prolonged life expectancy and an increasingly elderly population globally, more patients are evaluated for potential operative treatment for H&N cancers.

Despite the multidisciplinary approach to primary treatments as well as increased screening and monitoring, the incidence of loco-regional recurrence as well as second primary H&N cancers remains high. Repeated tumor resection was reported to be more effective in treating recurrent/second primary cancers than other therapies. Our department has aggressively adopted repeat resection with immediate microvascular reconstruction as the main curative option for treating recurrent/second primary H&N cancers, irrespective of the numbers of recurrence/second primary. The use of free flaps in reconstruction allows ‘like for like’ tissue replacement which was not achievable before the microsurgical era. It has also increased the possibilities to reconstruct larger defects caused by ablative surgery of tumors that previously were deemed unresectable. Several studies showed that cancer resections with immediate free flap reconstruction can be performed in elderly patients in an equally safe manner as compared to younger patients^4–6^. The most common reasons for a sequential free flap to the H&N area are tumor recurrence, occurrence of a second primary cancer or late complications due to previous surgery and/or radiotherapy such as fistula, trismus, or osteoradionecrosis. In the literature these unfavorable outcomes occur commonly^7,8^. However, the need for a third free flap is uncommon, few small to medium size case series have been reported^7,9,10^. To our knowledge, there is no study that described the impact of aging on operative results after a sequential third free flap to H&N.

Therefore, we describe our experience in the third episode of sequential free flap transfer in patients ≥ 65 years old in terms of medical, surgical, and flap-related complications. Further comparative analysis was performed, comparing these outcomes with patients < 65 years old.

## Results

### Demographics

Male is the dominant gender for the clinical presentation in this patient series. Overall 98.4% (124/126) of the patients were male and 1.6% were female (2/126). The mean age was 51.77 and 70.29 yeares in the younger and older group, respectively. The most common sites of primary malignancy were different between the groups, where buccal cancer was most common in the younger group (45.7%) and gum cancer in the older group (38.1%). There was no significant difference between groups regarding the purpose (functional recovery *vs*. tumor ablation) of the current reconstruction. Preoperative radiation was more common in the older group (38.1% *vs*. 18.1%, *p* = 0.041) whereas preoperative chemotherapy was more common in the younger group (69.5% *vs*. 42.9%, *p* = 0.025). Diabetes mellitus was more common in the older group (38.1% *vs*. 15.2%, *p* = 0.015). No other significant difference in the presence of different pre-existing disease could be elucidated. The baseline demographics for the two groups are provided in Table 1.

**Table 1:** Patients’ demographics.

	<65 years *n*(%)	≥65 years *n* (%)	*p* value
No.	105	21	
Age (yrs), Mean ± SD	51.8 ± 6.9	70.3 ± 5.1	<0.001
Gender			0.001
Male	105 (100)	19 (90.5)	
Female	0 (0)	2 (9.5)	
BMI^a^ (kg/m^2^), Mean ± SD	23.5 ± 3.9	23.1 ± 3.9	0.68
ASA^b^			0.43
ASA II	32 (30.5)	4 (19.0)	
ASA III	73 (69.5)	17 (81.0)	
Site of primary malignancy			0.001
Buccal	48 (45.7)	6 (28.6)	
Tongue	14 (13.3)	2 (9.5)	
Mandible	0 (0)	2 (9.5)	
Retromolar trigone	14 (13.3)	1 (4.8)	
Palate	6 (5.7)	0 (0)	
Lip	8 (7.6)	1 (4.8)	
Gum	12 (11.4)	8 (38.1)	
Hypopharynx	3 (2.9)	0 (0)	
Indication			0.62
Tumor	70 (66.7)	15 (71.4)	
Functional reconstruction	11 (10.5)	3 (14.3)	
Release contracture/trismus	16 (15.2)	1 (4.8)	
ORN^c^	8 (7.6)	2 (9.5)	
Preoperative radiation	19 (18.1)	8 (38.1)	0.04
Preoperative chemotherapy	73 (69.5)	9 (42.9)	0.03
Preexisting disease			
HTN^d^	22 (21.0)	7 (33.3)	0.22
Cardiovascular	3 (2.9)	2 (9.5)	0.15
Pulmonary	0 (0)	0 (0)	—
DM^e^	16 (15.2)	8 (38.1)	0.02
CVA^f^	3 (2.9)	2 (9.5)	0.15
Liver cirrhosis	8 (7.6)	4 (19.0)	0.10

^a^BMI, body mass index; ^b^ASA, American Society of anesthesiologists; ^c^ORN, osteoradionecrosis; ^d^HTN, hypertension; ^e^DM, diabetes mellitus; ^f^CVA, cerebral vascular accident.

### Operative data

The operative variables and flap selection are provided in Table 2. The most common new defect location was the buccal area. Recipient arteries in the ipsilateral neck were used for 41.9% in the younger group and 38.1% in the older group, with no statistical significance. We also noted a similar pattern across both groups with respect to the location of the recipient vein anastomosis (Table 2). There was no difference between the 2 groups regarding operation time, blood loss, ICU stay, hospital stay or mortality. The albumin levels were significantly lower in the older group (2.86 ± 0.41 *vs*. 3.18 ± 0.44, *p* = 0.006). There was no difference in flap selection for the first, second or third reconstruction. At all 3 occasions, anterolateral thigh flap (ALT) was the most commonly used flap. There were no cases of double free flap in the cohort.

**Table 2:** Operative variables.

	< 65 years *n* (%)	≥ 65 years *n* (%)	*p* value
Defect location, 3^rd^ surgery			0.12
Buccal	41 (39.0)	8 (40.0)	
Tongue	15 (14.3)	2 (10.0)	
Mandible	30 (28.6)	3 (15.0)	
Palate	3 (2.9)	0 (0)	
Lip	8 (7.6)	3 (15.0)	
Mouth floor	4 (3.8)	3 (15.0)	
Gum	3 (2.9)	0 (0)	
Hypopharynx	1 (1.0)	0 (0)	
Used flaps			
*First reconstruction*			0.684
ALT	68 (64.8)	11 (52.4)	
Fibula	18 (17.1)	6 (28.6)	
Forearm	6 (5.7)	2 (9.5)	
MSAP	1 (1.0)	0 (0)	
Other	12 (11.4)	2 (9.5)	
*Second reconstruction*			0.483
ALT	56 (53.3)	9 (42.9)	
Fibula	22 (21.0)	8 (38.1)	
Forearm	12 (11.4)	1 (4.8)	
MSAP	7 (6.7)	1 (4.8)	
Other	8 (7.6)	2 (9.5)	
*Third reconstruction*			0.535
ALT	33 (31.4)	8 (38.1)	
Fibula	19 (18.1)	5 (23.8)	
Forearm	13 (12.4)	0 (0)	
MSAP	10 (9.5)	2 (9.5)	
Other	30 (28.6)	6 (28.6)	
Duration between prior reconstruction (yrs)			
First and second, Mean ± SD	2.1 ± 1.7	2.0 ± 1.4	0.76
Second and third, Mean ± SD	1.7 ± 1.4	2.5 ± 2.3	0.14
Intraoperative variables			
Total operation time (min), Mean ± SD	558.4 ± 200.5	549.9 ± 205.1	0.86
Blood loss (mL), Mean ± SD	162.9 ± 161.9	115.2 ± 118.2	0.2
Albumin (g/dL), Mean ± SD	3.18 ± 0.44	2.86 ± 0.41	0.01

^a^Relative to the first surgery. ALT, anterolateral thigh; MSAP, medial sural artery perforator.

### Outcomes and complications

The overall flap success in this patient cohort was 94.4% (7 flap losses in 126 patients). Although statistical analysis showed no significant difference between groups regarding *overall* medical complications, the occurrence of medical complications was higher in the older group; 57.1% compared with 37.1% in the younger group. When looking at *specific* complications, cardiovascular complications were significantly more common in the older group (19% *vs*. 1.9%, *p* = 0.001). Similarly, delirium occurred more frequently in the older group compared with the younger group (23.8% *vs*. 6.7%, *p* = 0.023). There were no significant differences regarding surgical complications (Table 3).

**Table 3:** Outcomes and complications.

	< 65 years *n* (%)	≥ 65 years *n* (%)	*p* value
ICU stay (days), Mean ± SD	7.17 ± 5.78	6.52 ± 3.20	0.62
Hospital stay (days), Mean ± SD	28.71 ± 16.1	37.86 ± 49.44	0.411
Medical Complications			
Unplanned reintubation	7 (6.7)	0 (0)	0.223
LOS^a^ of MV^b^ (days), Mean ± SD	1.7 ± 1.5	1.8 ± 1.2	0.933
Interval between tracheostomy and de-cannulation	21.7 ± 13.9	18.0 ± 8.0	0.606
Permanent tracheostomy	26 (24.8)	6 (28.6)	0.785
Pulmonary	27 (25.7)	4 (19.0)	0.592
Cardiovascular	2 (1.9)	4 (19.0)	0.001*
Gastrointestinal	6 (5.7)	2 (9.5)	0.513
Renal failure	0 (0)	0 (0)	—
Delirium including Insomnia	59 (56.2)	15 (71.4)	0.231
Delirium	7 (6.7)	5 (23.8)	0.023^*^
Surgical Complications			
Recipient site infection	38 (36.2)	7 (33.3)	1
Flap related complications			
Re-exploration within 1 week	18 (17.1)	1 (4.8)	0.148
Total flap loss	7 (6.7)	0 (0)	0.223
Overall medical complications	39 (37.1)	12 (57.1)	0.096
Overall surgical complications	48 (45.7)	7 (33.3)	0.343

^a^LOS, length of stay; ^b^ MV, mechanical ventilation.

## Discussion

H&N cancer patients who require a third episode of repeat microvascular free tissue transfer represents a minority of cases currently. Nonetheless, the significance and size of this patient cohort is expected to increase in the near future and will then pose a regular challenge for both the ablative and reconstructive surgeon. This study, to the best of our knowledge consists of the greatest number of patients in the case series comprising longitudinal and sequential observations of surgical outcomes in a total of 126 patients receiving three sequential free flaps during separate occasions to the H&N area. This is also the first study to compare a younger and an older cohort over a 10-year period. Among them, most of the patients (67.5%) received repeated free flap reconstruction following resection of recurrent or second primary tumors. The indications for the remaining patients were to treat complications or functional deficits associated with previous radiotherapy and/or oncological and reconstructive surgery. Despite recent advances in diagnostic and therapeutic strategies, the prognosis after primary tumor resection for H&N cancers remains unsatisfactory because of the high incidence of recurrence and the occurrence of second primary cancer. The cumulative rate of recurrence/second primary cancer in H&N cancers is 17.9% at five years^11^. Therefore, repeated tumor resection with a sequential free flap reconstruction is not infrequently encountered in Taiwan. In our hospital, the selection criteria for treatment of recurrent or second primary cancers are the same as those used for primary cancers. The surgical indications in the elderly patients were similar to those in the younger patients. Our findings suggest that advanced age does not have an adverse effect on operative outcomes after third or more repeated resections followed by immediate free tissue transfer in patients with recurrent/second primary H&N cancers.

With increasing numbers of tumor resections, subsequent free flap reconstruction becomes increasingly more difficult. The flap success rate for all patients was 94.4%. This success rate is lower when compared with studies investigating surgical outcomes of first or second reconstructions, but comparable with other studies looking at third or more reconstructions^7,9,10^. The decline in flap success rate in cases with multiple previous reconstructions is multifactorial. Factors contributing to this phenomenon include scarring after previous surgeries, radiation-induced tissue injury, difficulties in vessel and flap selection, and malnutrition. In the third or more episodes of repeated microsurgical reconstruction, the recipient vessels in the neck that are most frequently used for microvascular anastomosis may be depleted or compromised by previous surgeries and irradiation. Possible strategies used to overcome these problems include the utilization of vein grafts for greater freedom of inset, alternative vessels for pedicle anastomosis outside the zone of injury, dissecting flaps with a longer vascular pedicle, and use of end to side anastomosis to major recipient vessels. The cephalic vein, transverse cervical vessels, thoracoacromial artery, and internal mammary vessels provide alternatives to conventional recipient vessels for the rare cases in which the neck is vessel depleted^12^.

The risk and benefit analysis of age and outcomes from the third repeated resection followed by immediate free tissue transfer should be assessed and justified. The occurrence of morbidity and postoperative complications are equally important and should both be considered in preoperative surgical decision making. Postoperative complications can lead to higher expenditure, prolonged hospital stay, decreased quality of life, especially in elderlies with lower functional reserve. In the study, there were no differences between the groups regarding overall medical and surgical outcomes, indicating performing a sequential third free flap in patients over 65 years equally safe when compared with outcomes in patients younger than 65 years. However, even though the overall medical outcomes did not differ, the incidence of cardiovascular complications and delirium were significantly more common in the older group.

H&N squamous cell carcinoma and presence of cardiovascular disease share two major risk factors - smoking and alcohol abuse. Previous studies showed a higher risk of cardiovascular complications after H&N surgery, especially with advanced age^13,14^. Similar outcome was observed in this study. This highlights the need for a thorough preoperative evaluation and optimization of patients who are planned to receive a major H&N surgery, especially in patients with advanced age.

Delirium is a feared postoperative condition as it is associated with higher rates of mortality, morbidity and longer hospitalization. It also confers a significant level of stress on the patient, relatives and health care providers^15,16^. In patients undergoing free flap reconstruction, delirium can pose a significant threat to the viability of the flap with unrestrained movement, especially after microvascular anastomosis. Patients with delirium often experience varying degrees of symptoms and suffer from disturbed consciousness and irritation, which may cause vascular injury to the flap pedicle. Previous studies have shown that longer operation times and free flap reconstruction are risk factors for postoperative delirium in elderly patients^17,18^. In the current study, there was an increased risk of postoperative delirium in the older group. However, the presence of delirium did not significantly increase mortality, morbidity or hospitalization time. One reason for this could be that the patients were nursed in a dedicated Microsurgery ICU during the first 7 post-operative days, as the first week is the most critical period for the establishment of peripheral circulation into the flap, and having intensive monitoring and care in Microsurgery ICU could minimize the potential anastomosis or pedicle injury due to delirium, hence reduce delirium related morbidity and mortality. Preoperative evaluation can also identify risk factors and predict the occurrence of delirium in some patients, and possibly even prevent its occurrence with greater awareness^19^. In the event of delirium, swift management with the assistance of a consulting geriatrician and/or psychiatrist is of the essence.

It is always important to strive for and complete an operation with efficiency. Efficient technical skill and operative logistics for intraoperative time reduction is essential, especially for older patients. Reduced intraoperative time could possibly decrease the risks of postoperative complications such as delirium and other medical conditions such as pressure ulcers and deep venous thrombosis. This can be achieved by two team approach both during the ablative part of the surgery (reconstructive team raises flap while ablative team resects tumor) and during the reconstructive part of the surgery (one reconstructive team finishes raising of the flap and closes the donor site while the second reconstructive team finds appropriate recipient vessels and performs the flap inset). Although not a routine preoperative investigation in the setting of multiple previous microvascular reconstructions, preoperative CT angiography to evaluate existing vessel options may facilitate the flow of the surgery and decrease operative time^20,21^.

Preoperative nutrition status evaluation plays an important role in post-operative recovery course. Clinical examination, anthropometric measurements and albumin level are common parameters that are used for nutrition status evaluation. Body Mass Index, an anthropometric measurement for nutritional status screening, showed no significant difference between the two groups, indirectly indicating similar nutritional status prior to the operation in these 2 groups. However, the albumin level in the older group was significantly lower. Previous studies have shown a decline in albumin levels is associated with increased age and drop in muscle mass, which likely explains the significantly lower levels in the older group of the current study^22,23^. Preoperative assessment of the patient’s nutritional status, and optimization when needed, is crucial in preventing foreseeable complications.

One must also remember that the patient’s biological age can differ significantly from the chronological age. As such, one of the limitations in studies of this nature is the possibility of selection bias, i.e. that the patient selection for surgery, consciously or subconsciously, is stricter the older the patient is. Other limitations are the retrospective nature of the study, most significantly the dependence of adequate journal notes. Finally, the study population is Asian, from an area with a very high incidence of head and neck cancer, and thus the generalizability of the results may be uncertain.

In conclusion, a sequential third free flap can be performed safely in patients who are more than 65 years of age, given reconstructive options such as vessel and flap selections were thoroughly evaluated preoperatively. A meticulous preoperative evaluation and optimization of the patient’s nutritional status and risk factors, especially regarding risk for cardiovascular events and delirium, is also crucial to achieve a successful outcome.

## Methods

### Patients

This study was approved by the institutional review board of Chang Gung Memorial Hospital (IRB number 105–1224 C). Informed consent was obtained from all subjects in the study, and institutional scientific regulations were followed throughout the study. The medical records of 3,524 patients who underwent microsurgical reconstruction after H&N tumor ablation at Chang Gung Memorial Hospital - Linkou Medical Center, Taiwan, between January 2004 and December 2013 were retrospectively reviewed. The data of consecutive patients who underwent 3 sequential free tissue transfer for recurrent/second primary cancer, osteoradionecrosis, trismus or scar contracture were collected in this study. Patients who met the following inclusion criteria were enrolled: (1) the first reconstruction was performed following curative resection of primary H&N cancer; (2) patients underwent 3 microvascular reconstructions in separate operations; (3) patients with complete follow-up and records. A total of 126 patients were enrolled in the study. These patients were divided into 2 age-related subgroups: patients aged less than 65 years and patients who were equal to or older than 65 years at the time of the operation.

### Patients’ data

Patient-related variables included age, gender, tumor stage, American Society of Anesthesiologist (ASA) classification, location of cancer, type of free flap and co-morbidities. Operative variables included operation time, albumin level, intraoperative blood loss, the need for tracheostomy, need for intraoperative blood transfusion more than 2 units, type of flap used in each episode of reconstruction, intensive care unit stay, and hospital stay. Following microsurgical free tissue transfer, all patients were closely monitored in a dedicated microsurgical intensive care unit for seven days.

### Definition of morbidity and mortality

The positive event for postoperative morbidity was defined as the patient having at least 1 of the major medical or surgical complications listed below. The occurrence of morbidity and mortality were restricted to the hospital stay. Medical morbidity was defined as any complication that resulted in organ dysfunction, including pulmonary, cardiac, and gastrointestinal complications, delirium, and renal failure. Unplanned postoperative reintubation was defined as a requirement for the placement of an endotracheal tube and mechanical or assisted ventilation because of the onset of airway compression or respiratory failure manifested by severe respiratory distress, hypoxia and/or hypercarbia within seven days following extubation. Surgical morbidities were defined as the presence of recipient-site infection and flap-related complications. Flap-related complications included partial or complete flap loss, re-exploration during the first postoperative week due to arterial or venous circulatory compromise. All deaths occurring within 30 days of surgery were considered surgical mortality.

### Statistical analysis

Characteristics of patients and clinical findings were stratified by age using 65 years of age as the cut-off point. Chi-square test, Fisher’s exact test, and Wilcoxon test were used for analysis where appropriate. The factors examined included gender, age, and other clinical factors. The statistical analysis was performed with SAS software version 9.1 (SAS Institute Inc., Cary, NC, USA). The level of significance for all P values was set at p < 0.05.

### Data availability

The datasets generated and analyzed during the current study are available from the corresponding author on reasonable request.
